# Regional anesthesia without opioid administration in mastectomy surgeries followed by breast reconstruction with implants: a randomized controlled study

**DOI:** 10.3325/cmj.2025.66.213

**Published:** 2025-06

**Authors:** Domagoj Eljuga, Rhea Marie Mužar, Ivo Jurišić, Jasminka Peršec, Ksenija Eljuga, Josip Jaman, Željka Roje, Krešimir Martić, Zlatko Vlajčić, Rado Žic

**Affiliations:** 1 Libertas International University, Zagreb, Croatia; 2 Department of Plastic and Reconstructive Surgery, Dubrava University Hospital, Zagreb, Croatia; 3 Department of Anesthesiology, Reanimatology and Intensive Care Medicine, Dubrava University Hospital, Zagreb, Croatia; 4 Nursing Department, Bjelovar University of Applied Sciences, Bjelovar, Croatia; 5 Department of Surgery, University of Zagreb School of Medicine, Zagreb, Croatia

## Abstract

**Aim:**

To compare regional anesthesia without opioid administration following subcutaneous mastectomy and breast reconstruction with implants in the pre-pectoral plane with general anesthesia in terms of pain relief, opioid consumption, and hospital stay duration.

**Methods:**

This randomized controlled study enrolled patients who underwent mastectomy with reconstruction of the breast either with permanent implants or tissue expander placement in the pre-pectoral plane. A total of 40 patients met the inclusion criteria. The regional anesthesia group (n = 20) received a pectoralis muscle block (PECS I), thoracic paravertebral block (TPVB), and serratus anterior muscle plane block (SAP) following a uniform protocol, and the control group (n = 20) underwent general anesthesia. Pain was assessed by using the numeric rating scale (NRS) from 30 minutes after surgery up to ten days postoperatively.

**Results:**

NRS pain scores were significantly higher in the regional anesthesia group, independent of the patients’ physical daily activity level, even up to 10 days after surgery. Opioid consumption and length of hospital stay did not differ significantly.

**Conclusion:**

Regional anesthesia using a combination of a TBVP, PECS I, and SAP has a long-lasting and satisfactory analgesic effect without the introduction of opioids. In the future, a novel gold standard protocol should be established that can be offered to every patient undergoing breast surgery.

Registration number: ISRCTN14701722.

Breast surgery is in growing demand not only for esthetic purposes but also in the field of surgical oncology. Breast cancer is the most common cancer in women worldwide, amounting to 25.8% of all cancers in 2020 (1). One of the main pillars of management of breast cancer is surgical therapy. Although breast-conserving therapy is preferred, mastectomy remains the mainstay therapy when a radical surgical intervention is indicated. The choice of therapy depends on the stage and type of carcinoma and is determined by a multidisciplinary team. Nowadays, integrated functional and esthetic breast reconstructions are commonly performed. Therefore, adequate perioperative management in reducing complication rates and pain control is crucial.

Pain, especially long-term pain, develops more often in women undergoing reconstructive surgical treatment, especially with implant-based reconstruction, rather than following mastectomy alone. The plane of implant placement is considered a risk factor, with submuscular implant placement being related to higher pain scores (2,3). Acute postoperative pain leads to higher opioid consumption, reduces life quality, prolongs hospital stay, and is a risk factor for the onset of chronic postoperative pain (2,3). Risk factors for persistent postsurgical pain were shown to be younger age, higher body mass index, anxiety, depression, diabetes mellitus, smoking, preoperative pain, moderate to severe acute postoperative pain, reoperation, radiotherapy, wound complications, and axillary lymph node dissection (4,5). The outcome not only depends on the surgeon and technique but also on the type of anesthesia. Regional anesthesia in combination with or without general anesthesia is increasingly used to reduce postoperative pain (2,3).

Regional anesthesia is often used in different surgical fields addressing regional nerves covering a defined dermatome (4,6,7). Sensory innervation of the breast is dermatomally organized – mainly originating from the anterolateral and medial branches of the thoracic intercostal nerves T2-6 and the supraclavicular nerves from the lower fibers of the cervical plexus, which supply the high upper and lateral parts of the breast  (4,6). Also, areas bordering the breast footprint, such as the pectoral muscles and the axillary region, must be included during anesthesia block design. Therefore, blocks in breast surgery to be considered are pectoralis muscle block (PECS I, PECS II), thoracal paravertebral block (TPVB), and serratus anterior muscle plane block (SAP).

The PECS block is applied between the pectoralis major and minor muscle. PECS I targets the medial and lateral pectoral nerves, providing anesthesia of the pectoralis major and minor muscles. PECS II blocks the lateral cutaneous branches of the intercostal nerve Th2-6. PVB aims to anaesthetize spinal roots in the paravertebral space dermatomes. SAP is given in the fascial plane superficial or deep to the serratus anterior muscle and provides analgesia to the long thoracic and thoracodorsal nerves. Thus, the serratus anterior muscles and latissimus dorsi muscle are covered (4). A uniform protocol has not yet been established for optimal results.

Our study aims to compare regional anesthesia without opioid administration following subcutaneous mastectomy and breast reconstruction with implants in the pre-pectoral plane with general anesthesia in terms of pain relief, opioid consumption, and hospital stay duration. Similar studies were conducted, but none of them have established a uniform protocol without the use of intraoperative opioids for oncological radical breast surgery with reconstruction using implants (3,6,8-11).

## Participants and methods

This randomized controlled study was conducted in the Department of Plastic, Reconstructive and Esthetic Surgery of University Hospital Dubrava. The study enrolled women undergoing skin-nipple-sparing or skin-sparing mastectomy with reconstruction of the breast either with permanent implants or tissue expander placement in the pre-pectoral plane. The surgeries were conducted between January 2022 and December 2023. The indication was either oncological or prophylactic based. A total of 364 patients underwent oncological breast surgery in the chosen period. Out of them, 95 were treated with either skin-nipple or skin-sparing mastectomy with implant based reconstruction. The inclusion criteria were age 18 to 90 years and the plane of implant placement above the pectoral muscle. Out of the 51 included patients, 11 refused to enter the study or were unable to cooperate because of age or mental state. The final sample involved 40 patients, and two were lost to follow-up due to failure to fill out the forms.

Patients were randomly assigned into groups using simple randomization, which was performed using a computer-generated sequence created in Microsoft Excel. The regional anesthesia group had 18 and the general anesthesia group had 20 patients. The mean age was 50 years (range 31-66 years). The study was approved by the Ethics Committee of Dubrava University Hospital. A written consent was obtained from all participants.

### Regional block technique

The application of blocks was standardized. Premedication consisted of 5 mg diazepam. Blocks were given unilaterally with multiple injection points – TBVB, PECS1, and SPB.

First, the patient was placed in the prone position, and landmarks for the TPVB – the transverse processes Th2, Th4, and Th6 were marked. The paravertebral region was identified with a low-frequency ultrasound (curved transducer). A mixture of 0.3%-0.5% levobupivacaine, dexamethasone, and adrenaline was administered in a dose of 0.1 mL/kg of bodyweight in three equal aliquotes at the Th2, Th4, and Th6 levels of the vertebrae.

Then, the patient was turned into a side position for the SPB. The block was applied in the midaxillary line at the level of the 5th rib by inserting the needle from posterior to anterior in the plane under the latissimus dorsi muscle and superficial to the serratus anterior muscle. A mixture of 0.25% levobupivacaine, dexamethasone, and adrenaline was given in a dose of 0.1 mL/kg of bodyweight.

For the PECS 1 block, the patient lay supine. The block was applied in the area between the pectoralis major and minor muscles. A mixture of 0.25% levobupivacaine, dexamethasone, and adrenaline was given in a dose of 0.1 mL/kg of bodyweight.

A total dose of dexamethasone of 8 mg and adrenaline of 200 μg was not exceeded. Adrenaline was not given to patients with atrial fibrillation and aortic stenosis. The efficacy of the block was confirmed by applying ice to the addressed dermatome. Half of the patients received sedation with sevoflurane and were given a laryngeal mask. The other half breathed spontaneously with an oxygen mask at a flow rate of 10 L/min. In addition, no opioids were given.

### Data collection and measurements

The primary outcomes were pain levels assessed using the numeric rating scale (NRS) in the morning, noon, and evening during physical activity and while resting. Assessments were made by the patient three times a day from 30 minutes up to 10 days after surgery.

Continuous variables such as age, body mass index, ASA status, the volume of the placed implant, and indication were collected from electronic health records. Consumption of opioids in the postoperative period and the length of hospital stay were also documented.

To minimize the bias caused by different operators, our study was conducted only in one center and one department, question forms were uniform for all patients and the medication offered to the patients was the same. All patients were treated in the same operating room and stayed in the same surgical ward with the same team of health care workers.

### Statistical methods

Data are presented as means and standard deviations. The normality of distribution of continuous variables was assessed using the Kolmogorov-Smirnov test. For independent samples, a *t* test was used to assess the differences in continuous variables between the groups. A χ^2^ test was used to test the differences between the groups in the length of hospital stay and opioid consumption. To test the differences between the groups in NRS, a mixed ANOVA test was used with a *post-hoc* Bonferroni test for the early postoperative period – 24 h, 7 days, and 10 days after surgery considering the level of activity as well. Statistical analysis was performed using the DATAtab Statistical Software (DATAtab Team, 2025; DATAtab: Online Statistics Calculator. DATAtab e.U. Graz, Austria).

## Results

There were no significant differences between the groups in the baseline age (*P* = 0.21), BMI (*P* = 0.498), ASA score (*P* = 0.828), and implant volume (*P* = 0.338) (unpaired *t* test; Table 1). NRS score in the early postoperative period was significantly higher in patients who underwent general anesthesia than in those who received regional anesthesia (mixed ANOVA: *P* < 0.001, *post-hoc* Bonferroni test *P* < 0.001).

**Table 1:** Patients’ characteristics in general anesthesia (GA) and regional anesthesia (RA) groups

		Minimum	Maximum	95% CI of the mean	Mean ± SD	p	Kolmogorov-Smirnov index
Age (year)	GA (n = 20)	32	66	44.14 - 51.66	47.9 ± 8.03	0.201	0.935
	RA (n = 18)	31	66	46.47 - 57.42	51.94 ± 11.01		
BMI (kg/m^2^)	GA (n = 20)	18.37	34.25	22.78 - 26.98	24.88 ± 4.48	0.498	0.851
	RA (n = 18)	20.24	38.58	23.6 - 28.17	25.89 ± 4.59		
Volume implant (mL)	GA (n = 20)	240	545	350.6 - 441.9	396.25 ± 97.55	0.338	0.807
	RA (n = 18)	285	575	386.62 - 461.16	423.89 ± 74.94		
Duration of hospitalization (days)	GA (n = 20)	4	10	7.17 - 8.73	7.95 ± 1.67	0.993	0.35
	RA (n = 18)	5	13	6.85 - 9.04	7.94 ± 2.21		
ASA score	GA (n = 20)	1	2	3	1.58 - 2.12	1.85 ± 0.59	0.828
	RA (n = 18)	1	2	3	1.46 - 2.1	1.78 ± 0.65	

*Abbreviations: BMI – body mass index, ASA – American Society of Anesthesiologists, CI – confidence interval, SD – standard deviation.

**Figure 1:**
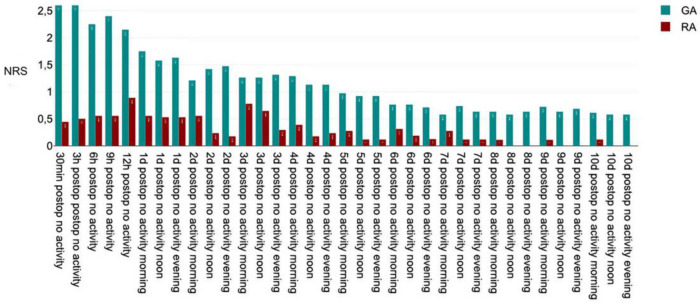
Numeric rating scale (NRS) scores throughout the 10 days at rest. GA – general anesthesia; RA – regional anesthesia.

The NRS at rest 24 hours after surgery was significantly higher in the general anesthesia group (mixed ANOVA: *P* = 0.004, *post-hoc* Bonferroni test: *P* = 0.004). Significantly higher values were also documented in the general anesthesia group at rest 7 and 10 days after surgery, with daily measurements being conducted in the morning, noon, and evening (7 days mixed ANOVA: *P* = 0.015, *post-hoc* Bonferroni test *P* = 0.015, 10 days mixed ANOVA: *P* = 0.015, *post-hoc* Bonferroni test *P* = 0.015) (Figure 1).

**Figure 2:**
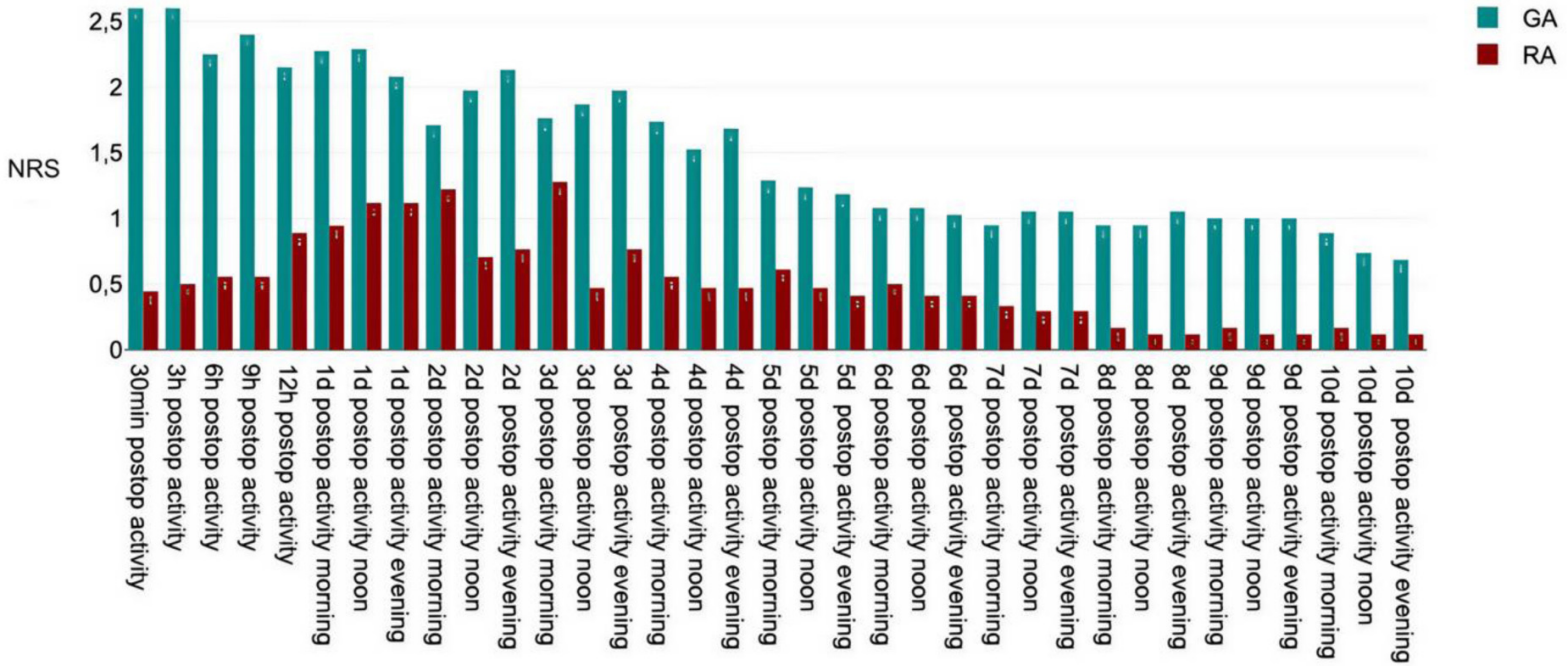
Numeric rating scale (NRS) scores throughout 10 days with daily activity. GA – general anesthesia; RA – regional anesthesia.

Similarly, the NRS of patients performing normal daily activity 24 h, 7 days, and 10 days after surgery, with daily measurements conducted in the morning, noon, and evening, were also significantly higher in the general anesthesia group (24-h mixed ANOVA: *P* = 0.007, *post-hoc* Bonferroni test *P* = 0.007, 7d mixed ANOVA: *P* = 0.003, *post-hoc* Bonferroni test *P* = 0.003, 10d mixed ANOVA: *P* = 0.002, *post-hoc* Bonferroni test *P* = 0.002) (Figure 2). Table 2 shows an overview of the NRS values in the early postoperative period, after 24 hours, 3 days, and 10 days.

**Table 2:** Numeric rating scale (NRS) scores throughout 10 days

	NRS (mean ± SD)				
Time point	regional anesthesia		general anesthesia		P value
	no activity	activity	no activity	activity	
30 min	0.44 ± 0.92	0.44 ± 0.92	2.6 ± 2.66	2.6 ± 2.66	<0.001
6 h	0.56 ± 0.92	0.56 ± 0.92	2.25 ± 1.71	2.25 ± 1.71	<0.001
1 day morning	0.56 ± 0.92	0.94 ± 1.26	1.75 ± 1.62	2.28 ± 1.62	0.004
5 day morning	0.28 ± 0.83	0.61 ± 0.98	0.97 ± 0.79	1.29 ± 0.87	0.001
10 day morning	0.12 ± 0.49	0.17 ± 0.51	0.61 ± 0.92	0.89 ± 1.28	0.002

There was no significant difference in opioid consumption (*P* = 0.11, χ^2^ test, with a middle effect size of Cramér’s V = 0.26) or the length of hospital stay throughout the ten days (*t* test; GA mean 7.95 days, RA mean 7.94 days, *P* = 0.993).

## Discussion

In this study, pain scores were significantly higher when using a combination of TVB, PECS I, and SAP block compared with general anesthesia, independent of the patients’ physical daily activity level, and even up to 10 days after surgery.

Nowadays, surgical therapy in breast cancer unites oncological, esthetic, and functional aspects to achieve optimal results. But the quality of life might be compromised if postoperative pain in the short and long term is not optimally managed. Several studies following breast oncological surgery reported acute postoperative pain in 10%-20% of patients and chronic pain after 3 years in up to 50% of the patients (6,12).

Pain also leads to a greater need for analgesic therapy. Prolonged narcotics use is not recommended because they are associated with side effects such as altered mental status, nausea, constipation, respiratory depression, and addiction.

There is still no standard protocol for minimizing postoperative pain in reconstructive breast surgery, both in the short and long term. In the study by Wong et al, analgesic support with non-steroidal pain killers and intraoperative local anesthetic blocks or, depending on the indication, PECS I and serratus anterior plane block, reduced narcotic pain medication use (7). We aimed to improve the anesthetic protocol by Wong et al (7). Several surgical fields already use regional blocks as a gold standard, but the ideal protocol for breast surgery is still evolving.

A meta-analysis that examined the most effective regional anesthesia technique for breast cancer surgery showed reduced acute pain and postoperative nausea and vomiting in patients who received a thoracic paravertebral block, pectoral block, serratus anterior plane block, errector spine plane block, and intercostal nerve block (12).

Several reports have shown the benefits of paravertebral blocks in breast surgery. Prospective and retrospective studies described significantly lower pain in breast reconstruction surgery when PVB was administered (2,8,9,12-14). Sforza et al found that regional block anesthesia using the S-PECS block for sub-glandular breast augmentation significantly reduced pain scores compared with general anesthesia (15). A meta-analysis of PECS block in oncological breast surgery showed effective pain reduction in the first 24 hours following surgery compared with general anesthesia only (2). Some even consider the PECS block a first-line regional anesthetic (12). In some studies, the intraoperative intercostal block showed reduced hospital stay and a lower need for narcotic analgesia compared with no additional block anesthesia (9,12).

However, adding supplementary blocks to the PVB offers the advantage of a broader operative field, as demonstrated in our study. Significantly lower NRS pain scores were reported regardless of patients’ daily activity levels. We attribute this to the coverage of the axillary region and the puncture sites for drainage placement within the dermatomes anesthetized by the additional serratus anterior plane block. This approach facilitates earlier, pain-free mobilization of patients.

A lack of significant differences in the length of hospital stay might be explained by our hospital’s postoperative protocol, with the volume of drainage being one of the main factors in determining the discharge date, not just the patient's subjective feelings. Other studies have documented a decreased length of stay when regional anesthesia was applied (9,12).

A lack of significant difference in opioid consumption in our study could be attributed to the already low baseline use of narcotic agents in our hospital. However, multiple studies from different countries have shown a significant reduction in opioid requirements when regional anesthesia was used (7,10,11).

Complications of regional anesthesia should also be pointed out. No adverse effects of regional anesthesia were noted in our study. Still, most frequently described complications are pneumothorax, local site infection, hemodynamic instability, and diffusion of local anesthetic out of the areas required to numb. We observed mental discomfort in some patients due to the wide-awake application. This complex issue can be addressed by a clear explanation before the procedure. As in our study, regional block anesthesia is applied by an experienced anesthesiologist to minimize the complication rate.

The single-center nature of the study limited the generalizability of the findings. Possible bias introduced by different teams of surgeons and anesthesiologists was offset by the introduction of standardized protocols. The benefits of regional anesthesia in breast surgery in the retro-pectoral plane should be further investigated. An interesting study has also shown promising results in microsurgical breast reconstruction with the DIEP flap, which could be investigated with a more extensive patient cohort (8).

In conclusion, in our study, the administration of combined regional blocks ensured a significantly longer analgesic effect without the introduction of opioids in a well-defined group compared with general anesthesia with opioids. Our findings contribute to the pool of findings on the use of different regional blocks used in breast surgery. Regional anesthesia is a field to be further explored, with an aim to establish a novel gold standard protocol that can be offered to every patient undergoing breast surgery.
